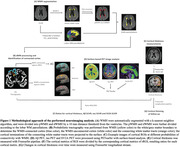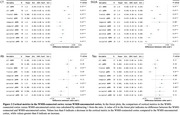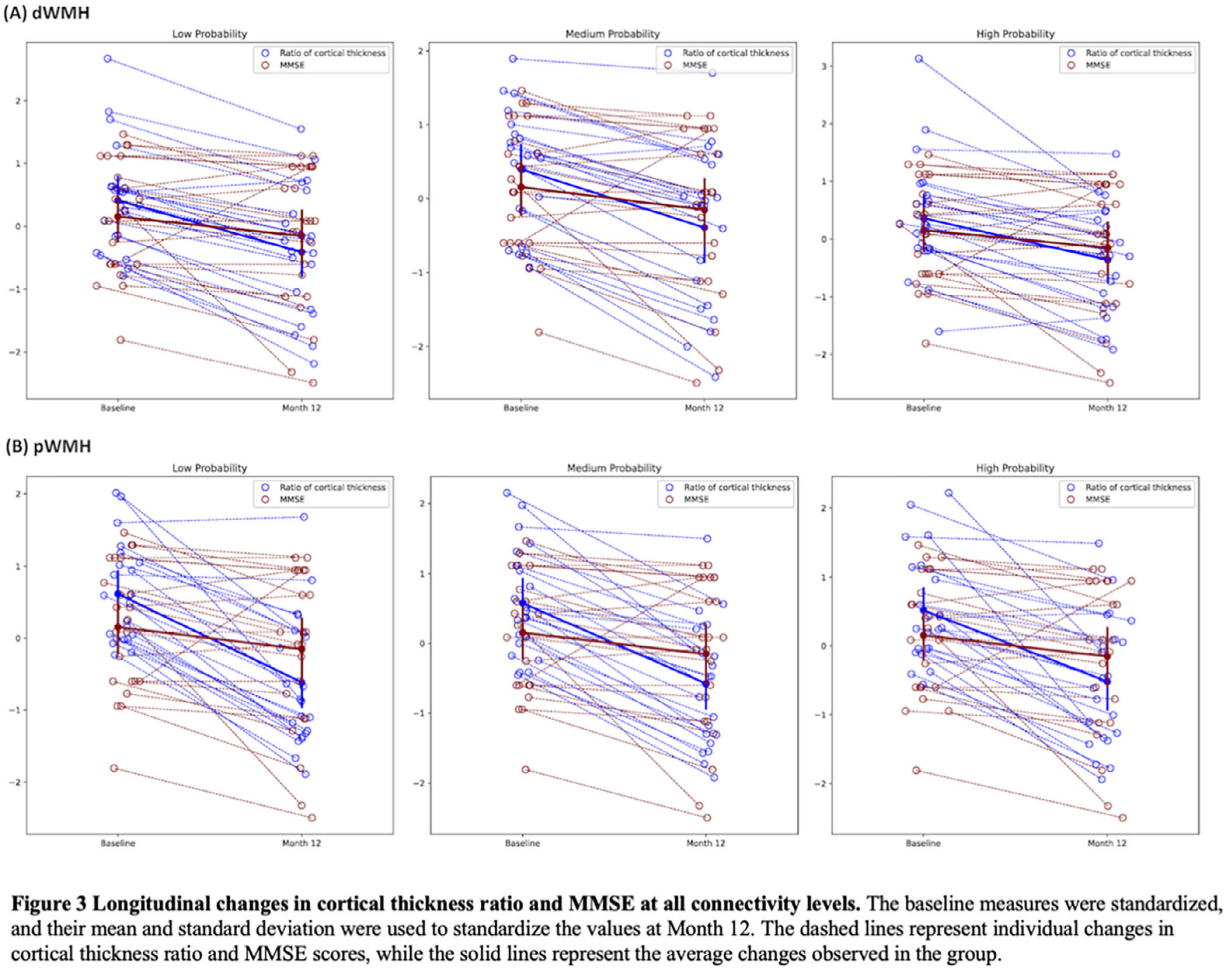# Linking White Matter Hyperintensities to Regional Cortical Thinning, Amyloid Deposition, and Synaptic Density Loss in Alzheimer's Disease

**DOI:** 10.1002/alz.085669

**Published:** 2025-01-09

**Authors:** Junfang Zhang, Binyin Li

**Affiliations:** ^1^ Ruijin Hospital affiliated with Shanghai Jiao Tong University School of Medicine, Shanghai, Shanghai China; ^2^ Ruijin Hospital affiliated to Shanghai Jiaotong University School of Medicine, Shanghai China

## Abstract

**Background:**

White matter hyperintensities (WMH) were reported to contribute to the thinning of regional cortex connected to WMH in cerebral small vessel disease. However, the relationship between WMH and regional changes in WMH‐connected cortex in Alzheimer’s disease (AD) remains unclear. The objective of this study is to investigate the association between WMH and regional cortical thickness, amyloid and tau deposition, and synaptic density changes in the WMH‐connected cortex.

**Method:**

In the cross‐sectional analysis, we included 107 participants (59 AD, 27 mild cognitive impairment, and 21 cognitively normal controls) who tested positive for beta‐amyloid (Aβ) using amyloid‐PET. All the participants had T1, fluid‐attenuated inversion recovery (FLAIR), multi‐shell diffusion MRI, tau‐PET, and cognitive assessment. A subset (n=33) had synaptic vesicle glycoprotein 2 A (SV2A) PET to assess synaptic density. We segmented WMH on FLAIR images. The cortex connected to WMH was identified using probabilistic tractography. We measured the cortical thickness, amyloid and tau deposition, and synaptic density based on T1 images, amyloid‐PET, tau‐PET, and SV2A‐PET. A subset of participants (n=23) underwent a follow‐up multimodal MRI after 12 months. Changes in cortical thickness were measured using the longitudinal stream within FreeSurfer. (Figure 1)

**Result:**

We found that WMH‐connected cortex showed significantly lower thickness and synaptic density than WMH‐unconnected cortex (all *p‐corrected*<0.001). Besides, higher levels of Aβ deposition in frontal (ratio = 1.045, *p*<0.001), temporal (ratio=1.045, *p*=0.002), and parietal (ratio=1.054, *p*<0.001) deep WMH (dWMH)‐connected cortex, and higher levels of tau deposition in parietal dWMH‐connected cortex (ratio=1.050, *p*<0.001) were found, compared to the corresponding WMH‐unconnected cortex. (Figure 2) After adjusting for age, education years, and volume of WMH, higher Aβ ratio in the total dWMH‐connected cortex was significantly related to lower cognitive score (linear regression: *β*=‐37.8, *p‐corrected*=0.040). Lastly, the cortical thickness of WMH‐connected cortex reduced more than WMH‐unconnected cortex over 12 months (all *p‐corrected* <0.001, Figure 3).

**Conclusion:**

We revealed that WMH are related to more severe regional cortical degeneration as measured by thickness, Aβ, tau, and synaptic density in the WMH‐connected cortex in AD. Our results suggest that WMH are likely to be associated with AD‐intrinsic processes of degeneration, in addition to vascular mechanism.